# Mental health care access in rural communities amid the expansion of telehealth

**DOI:** 10.1093/haschl/qxag241

**Published:** 2026-09-04

**Authors:** Anja Gruber, Anders Van Sandt, Scott Loveridge, Craig Carpenter, Cris Meier

**Affiliations:** Department of Agricultural and Applied Economics, University of Wyoming, Laramie, WY 82071, United States; Department of Agricultural and Resource Economics, Colorado State University, Fort Collins, CO 80521, United States; Department of Agricultural, Food, and Resource Economics, Michigan State University, East Lansing, MI 48824, United States; Department of Agricultural, Food, and Resource Economics, Michigan State University, East Lansing, MI 48824, United States; Department of Social Work, Utah State University, Logan, UT 84322, United States

**Keywords:** mental health, provider shortages, telehealth, equitable access to health care

## Abstract

**Introduction:**

Compared to metropolitan residents, rural residents face disparities in access to local mental health providers that are even larger than for other health care services. Telehealth appears to be a solution: patients should be able to more easily access providers from metro areas, reducing disparities in access. We consider how provider composition in telehealth or changes in provider behavior enabled by telehealth are associated with access to telehealth.

**Methods:**

We present descriptive evidence from multivariate regressions using National Health Interview Survey and Medical Expenditure Panel Survey data.

**Results:**

In recent years, rural and publicly insured patients have become relatively less likely to see a mental health provider. Simultaneously, access to any telehealth services is 8-9 percentage points lower for non-metro residents, even after accounting for potential differences in mental health care utilization and internet access. We also show that self-payment is associated with telehealth utilization.

**Conclusion:**

We caution that telehealth in mental health may disproportionately benefit patients with higher insurance reimbursement or higher probability of self-pay and thus not reduce the mental health access gap for rural patients. Expansions in telehealth need to be combined with policies to promote equitable reimbursement and reduce barriers for providers to bill insurance.

## Introduction

Relative to urban areas, rural counties in the United States face structural challenges that have long translated into worse access to health care and poorer health outcomes. These challenges include older and shrinking populations, persistent provider shortages, higher rates of uninsurance and public insurance—which translate into lower provider reimbursement—lack of economies of scale, greater travel distances, and many others.^1^

Today, increased access to remote care via phone or video conferencing (hereafter referred to as telehealth) is often cited as a one-size-fits-all solution for connecting rural residents with the care they need.^2^ The expansion of broadband,^3^ a shift in preferences as the result of the COVID-19 pandemic,^4^ and state and federal coverage and payment parity policies have contributed to widespread increases in the availability and utilization of telehealth.^5-7^ Prior to 2020, 22 states implemented telehealth coverage parity for private insurers; in March 2020, Medicare implemented universal payment parity. While this policy was never put in place permanently, it was extended through December 2027.^8^ Many states also implemented Medicaid telehealth parity the same year.

Following a rapid increase in telehealth usage across medical services in 2020, telehealth treatments remain most widely used in psychotherapy or mental health counseling (hereafter referred to as mental health treatments), where it is perhaps most easily implemented. In 2021, 2022, and 2023, roughly 50% of mental health appointments were conducted via telehealth (authors' calculations, Medical Expenditure Panel Survey [MEPS] data).

**Figure 1.**
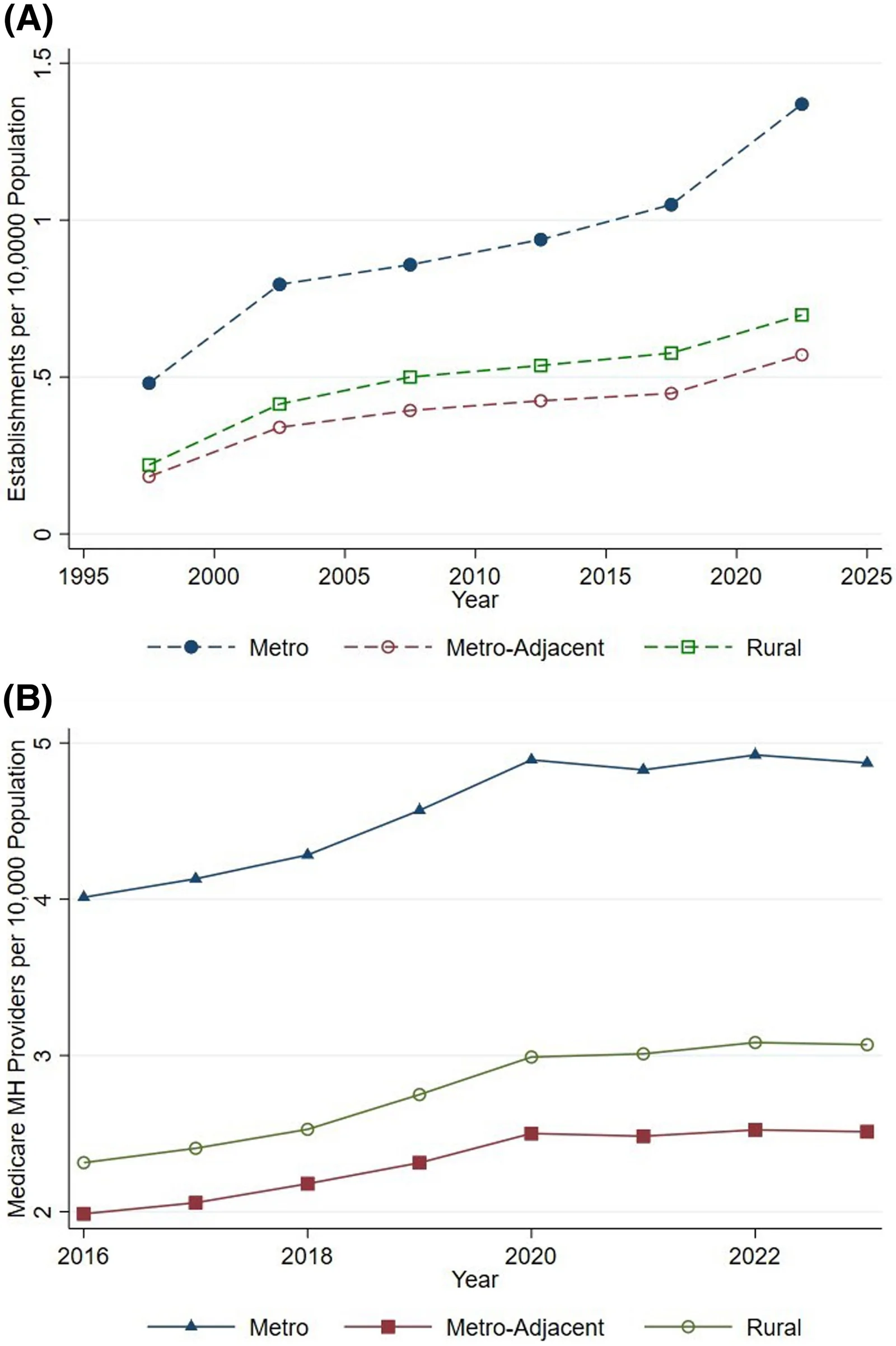
Access to mental health care by rurality. (A) Mental health care establishments per 10 000 population and (B) Medicare mental health providers per 10 000 population. The figure provides a summary of access to mental health care in metro, metro-adjacent, and rural counties as defined by the USDA 2019 rural–urban continuum codes 1-3, 4, 6, and 8, and 5, 7, and 9. Panel 1 shows the number of mental health physician and practitioner establishments (NAICS 621112 and 621330) per 10 000 population over time. This research was performed at a Federal Statistical Research Data Center under FSRDC Project Number 2716, clearance request #12887. Panel 2 shows the number of Medicare-enrolled mental health care providers per 10 000 population, calculated from 2016 to 2023 CMS Medicare provider data.

At the same time, the rural–urban gap in access to care is particularly large in mental health care. Among rural counties, 96% are designated Mental Health Professional Shortage Areas (MHPSA), making up 85% of counties with this designation. Provider-to-population ratios are much lower for all mental health care-related occupations, including psychiatrists, psychologists, counselors, psychiatric nurse practitioners, and social workers.^9^ Panel (A) of Figure 1 shows that while the number of mental health establishments per 10 000 population has increased in both rural (USDA rural–urban continuum codes 5, 7, and 9) and metro counties (USDA rural–urban continuum codes 1-3), the discrepancy is large and continues to widen, especially in recent years. Similarly, panel (B) shows that the number of Medicare-registered mental health providers per 10 000 population is increasing across the country, but the discrepancy between rural and metro provider numbers is large.

The rapid and widespread expansion of telehealth use in mental health treatment raises important questions. The logic of expanding telehealth is straightforward: It enables patients to access providers outside their area of residence and thus increases the number of providers accessible to them. Equally important, but much less considered, are the potential consequences for provider availability to individual patients. In mental health, where there are particularly widespread provider shortages^10^ and acceptance rate for patients with lower insurance reimbursement are lower,^11-13^ there is a possibility that providers who are less willing to accept patients with lower reimbursement are more likely to provide telehealth, or that telehealth allows providers to become more selective. If that is the case, telehealth might improve access only for patients with insurances with higher provider reimbursement or for patients who are able to self-pay.

In this article, we provide descriptive evidence aimed to investigate whether telehealth expansion is associated with improved access to mental health services. We show how the gap in mental health care utilization has evolved in recent years. We also show how rural status is correlated with the use of telehealth for individuals with and without mental health visits and estimate multivariate regressions to test how socio-economic and demographic characteristics correlate with mental health care utilization and telehealth utilization. Finally, we investigate a potential relationship between self-pay and telehealth use.

Our study is descriptive but nonetheless illustrates the importance of future research that can identify the causal impact of telehealth expansion on access to care for different groups of patients. We caution from viewing telehealth as a one-size-fits-all solution to promote equitable access to care. Especially in mental health care, where provider shortages are widespread, policies that promote telehealth adoption need to be coupled with efforts that increase providers' willingness to serve both public and privately insured patients and efforts to increase utilization among less educated and lower-income patients.

## Data and empirical strategy

Public data sources have begun measuring telehealth usage only since the COVID-19 pandemic, making it difficult to draw conclusions on the relationship between telehealth and access to care. We rely on data from the National Health Interview Survey (NHIS) and the MEPS, 2 of the largest nationally representative US health surveys. While the NHIS provides variables for telehealth and mental health provider utilization in the last year, it does not allow us to identify telehealth use specifically for mental health treatments. With MEPS data, we can assess telehealth use at mental health visits, but we are not able to compare rural and urban residents. Thus, we rely on cumulative evidence from both data sources.

We use annual, cross-sectional, individual-level data from the NHIS to estimate multivariate linear probability models (LPMs). These models identify the demographic and socio-economic characteristics that predict whether an individual saw a mental health care provider within the last year, and whether an individual utilized telehealth within the last year. Key variables of interest are not consistently available in the NHIS, restricting us to use years 2020-2024 for telehealth utilization, 2020-2024 for rural and metro categories, and years 2015-2024 but excluding 2019 for mental health care utilization. Regressions are weighted using survey weights, and robust standard errors are reported. Based on our models, we calculate year-specific adjusted differences in the predicted probability of mental health professional use between individuals in non-metro and metro areas and individuals with and without public insurance, based on the fitted regression model and averaged over the observed covariate distribution.

We then exploit 2020-2023 MEPS data on individual office-based medical provider visits and outpatient visits. To do so, we merge MEPS individual patient demographic information with each of the “Visits” files and retain all patients with at least one psychotherapy or mental health counseling service. We collapse the data to the individual patient level and again estimate multivariate linear regressions to determine the association of self-payment with telehealth usage for mental health services; because the dependent variable in this analysis is the share of a patient's mental health visits conducted via telehealth rather than a binary indicator, we describe these estimates as linear regressions rather than LPMs. Again, regressions are weighted using survey weights and robust standard errors were computed.

## Results

**Figure 2.**
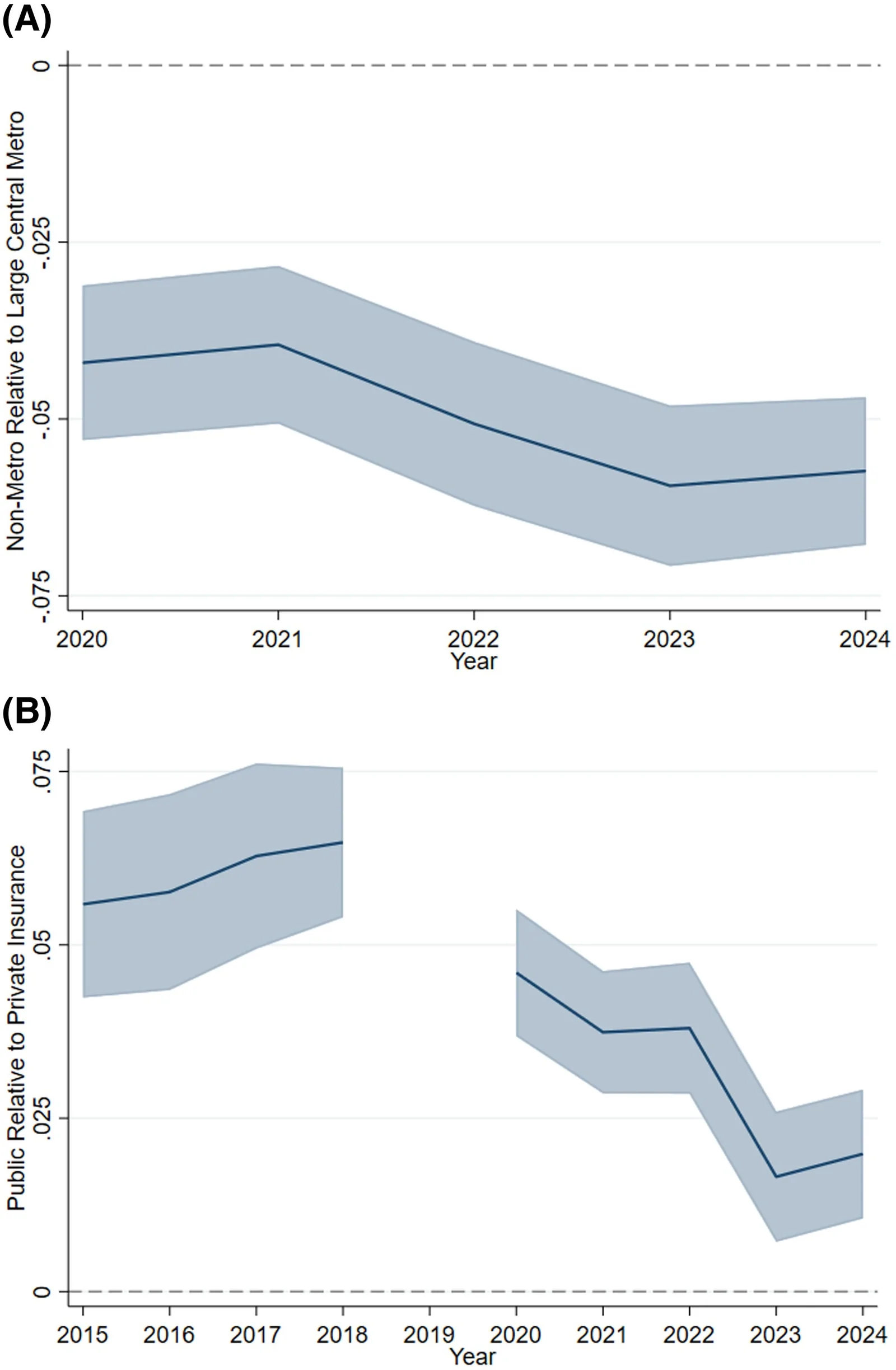
Regression-adjusted gap in probability of seeing a mental health professional. (A) Rural patients and (B) publicly insured patients. The figure shows the regression-adjusted gap in the probability of having seen a mental health professional in the last 12 months, using data from the 2015-2024 NHIS survey. 95% confidence intervals are displayed. Years and covariates are based on the availability of variables. Appendix Table S2 provides full regression tables. Rural–urban classifications are available only since 2020 and the outcome variable is not available in 2019. Therefore, the results are based on 2 separate regressions. Regressions are weighted using survey weights. Standard errors are robust.

Figure 2 shows the regression-adjusted gap in the probability of having seen a mental health professional in the last 12 months for non-metro relative to large central metro individuals in panel (A) and for individuals with public relative to private health insurance in panel (B). Appendix Table S1 provides the means of our NHIS sample. The full regression results for each regression and underlying sample are shown in Appendix Table S2. Variables and years included in each regression are limited based on availability in the NHIS survey. We note that from 2020-2024, on average 12% of individuals saw a mental health professional in the last year, and 28% of individuals had at least one telehealth appointment in the last year.

We show that even after controlling for differences in self-reported mental health and demographic and socio-economic characteristics, non-metro patients are roughly 5 percentage points less likely than large central metro residents to have seen a mental health provider in the last year. This gap has grown since 2020. On the other hand, publicly insured patients were roughly 5 percentage points more likely than privately insured patients to have seen a mental health provider in 2015, but this gap has more than halved since. Note that coefficients are relative to the omitted category, “2015, no public health insurance.” We conduct a formal joint test and find that the difference in the probability of seeing a mental health professional between individuals in non-metro and metro areas and individuals with and without public insurance varies significantly across years (*P* < .0001).

Table 1, column 1 shows the results for the individual-level linear probability model predicting the use of telehealth in the last year. In terms of rural access, first, individuals in large central metro areas who saw a mental health professional are 33.1 percentage points more likely to have had a telehealth appointment in the last year (0.331). Second, non-metro residence is associated with an 8.3 percentage point lower probability of having had a telehealth appointment relative to large central metro residence (−0.083). The interaction term (0.233) gives the corresponding estimate for non-metro residents who saw a mental health professional, and the difference between the 2 estimates therefore serves as a test of disparate impact: we formally reject their equality, indicating that the telehealth premium associated with seeing a mental health professional is 9.9 percentage points smaller in non-metro areas. Taken together with the direct non-metro coefficient, non-metro residents who saw a mental health professional are roughly 18 percentage points less likely to have used telehealth than otherwise similar large central metro residents who did so. Relative to privately insured patients, Medicare and Medicare Advantage beneficiaries are more likely to have had a telehealth appointment. Uninsured patients are the least likely to have had a telehealth appointment. Individuals who note that they have a usual place for care are 9 percentage points more likely to have used telehealth. Women, married patients, higher-income patients, and patients with higher levels of education are more likely to have had a telehealth appointment. We also show that relative to large central metro areas, individuals in non-metro areas are 8 percentage points less likely to have had a telehealth appointment in the last year.

**Table 1.** Predicting the use of telehealth services.

	Dependent variable: had a telehealth appointment in the last year (1 = yes, 0 = no)			
	Coeff.	Std. err.	Coeff.	Std. err.
	(1)		(2)	
Saw an MH professional last year	0.3311***	(0.0050)	0.3643***	(0.0069)
Saw an MH prof, last year # non-metro	0.2325***	(0.0132)	0.2666***	(0.0183)
Has home internet access			0.0592***	(0.0108)
No insurance *(excluded: other insurances)*	−0.0815***	(0.0043)	−0.0777***	(0.0065)
Medicare FFS insurance	0.0398***	(0.0057)	0.0297***	(0.0085)
Medicare advantage insurance	0.0262***	(0.0050)	0.0385***	(0.0074)
Medicaid insurance	0.0161***	(0.0049)	0.0062	(0.0071)
Has a usual place for care	0.0915***	(0.0035)	0.0730***	(0.0053)
Age 31-50 *(excluded cat: 18-30)*	0.0430***	(0.0039)	0.0507***	(0.0055)
Age 51-65	0.0571***	(0.0042)	0.0599***	(0.0061)
Age 65 or older	0.0288***	(0.0063)	0.0211**	(0.0093)
Female	0.0483***	(0.0026)	0.0524***	(0.0038)
Married	0.0156***	(0.0027)	0.0102**	(0.0040)
Black *(excluded race: White)*	−0.0144***	(0.0043)	−0.0093	(0.0065)
Asian	−0.0452***	(0.0055)	−0.0407***	(0.0082)
All other races	−0.0021	(0.0050)	0.0000	(0.0073)
Hispanic	−0.0177***	(0.0043)	−0.0280***	(0.0061)
HS degree *(excluded: no HS degree)*	−0.0045	(0.0050)	−0.0138*	(0.0076)
At least some college	−0.0006	(0.0053)	−0.0095	(0.0080)
Income: Pov threshold ratio 1-2 *(excluded: <1)*	0.0081	(0.0051)	0.0052	(0.0076)
Income: Pov threshold ratio 2-3	0.0178***	(0.0046)	0.0256***	(0.0072)
Income: Pov threshold ratio >3	0.0663***	(0.0046)	0.0704***	(0.0071)
Non-metro (excluded cat: large central metro)	−0.0828***	(0.0041)	−0.0846***	(0.0060)
Small-to-medium metro	−0.0498***	(0.0034)	−0.0565***	(0.0049)
Large fringe metro	−0.0101***	(0.0037)	−0.0097*	(0.0054)
2021 *(excluded year: 2020)*	0.1989***	(0.0040)		
2022	0.1262***	(0.0039)	0.0000	(.)
2023	0.0897***	(0.0037)	−0.0197***	(0.0052)
2024	0.0698***	(0.0037)	−0.0402***	(0.0052)
Midwest *(excluded region: Northeast)*	−0.0388***	(0.0042)	−0.0355***	(0.0061)
South	−0.0128***	(0.0039)	−0.0036	(0.0057)
West	0.0433***	(0.0043)	0.0608***	(0.0063)
Constant	−0.0301***	(0.0083)	0.0344**	(0.0162)
Observations	150 852		69 516	
Adjusted *R*-squared	0.126		0.126	

Table reports coefficients from an individual-level linear probability model predicting use of telehealth in the last year using data from the 2020-2024 NHIS survey. Regressions are weighted using survey weights. Standard errors are robust.

^*^ *P* < .10, **P < .05, ***P < .01.

We add a variable to control for home access to the internet in column (2) of Table 1. This variable is available only since 2022, restricting this analysis to a smaller sample. Nonetheless, if differences in internet access are the driver of rural–urban telehealth usage gaps or gaps for individuals with lower socio-economic status, inclusion of the home-internet control should reduce the explanatory power of these other variables. We show that this is not the case. Conditional on access to the internet at home, the difference in the probability of having had a telehealth appointment in non-metro relative to large central metro areas remains roughly 8 percentage points. The estimated non-metro shortfall among individuals who saw a mental health provider is also nearly unchanged, at 9.8 percentage points. However, conditional on having home access to the internet, the coefficients on Black and higher income are no longer statistically significant, indicating that lower access to telehealth for Black and lower-income patients is related to differences in access to technology.

Table 2 shows the results of our multivariate regression using MEPS data. Means of patients with at least one mental health appointment are shown in Appendix Table S3. The analogous NHIS regressions predicting the use of a mental health professional are reported in Appendix Table S2. We find that 29% of mental health appointments in our data took place via telehealth. Of all patients with at least one mental health appointment, 13% fully self-paid, whereas 46% of patients had their treatment fully covered by insurance (no self-payment). Among patients who receive mental health treatment, self-payment (or out of pocket payment) can help understand conditions associated with differences in access to telehealth. Individuals who self-pay for all of their treatment have, on average, a 5.4 percentage point higher share of treatments conducted online compared to individuals who do not fully self-pay.

**Table 2.** Use of telehealth services at mental health appointments is associated with higher rates of self-pay.

	Dependent variable: share of MH visits via telehealth (0-1)	
	Coeff.	Std. err.
Fully self-paid	0.054***	(0.019)
Number of MH visits	−0.001***	(0.000)
Share of MH visits with psychologist	0.022	(0.022)
Share of MH visits with psychiatrist	0.0305	(0.020)
Share of MH visits with counselor	0.032*	(0.018)
Share of MH visits office-based	−0.217***	(0.026)
No health insurance *(excluded: other insurances)*	−0.068*	(0.040)
Medicare FFS insured	−0.025	(0.025)
Medicare advantage insured	0.011	(0.029)
Medicaid insured	−0.077***	(0.019)
Age 31-50 *(excluded cat: 18-30)*	−0.018	(0.016)
Age 51-65	−0.050***	(0.018)
Age 65 or older	−0.105***	(0.029)
Female	0.007	(0.013)
Married	−0.022*	(0.013)
Black *(excluded race: White)*	0.082***	(0.020)
Asian	−0.079	(0.055)
All other races	0.006	(0.023)
Hispanic	0.023	(0.018)
HS degree *(excluded: No HS Degree)*	0.037	(0.024)
At least some college	0.071***	(0.022)
Near poor *(excluded: Pov threshold ratio <1)*	−0.004	(0.031)
Low income	−0.003	(0.024)
Middle income	0.025	(0.021)
High income	0.041*	(0.021)
2021 *(excluded year: 2020)*	0.264***	(0.014)
2022	0.274***	(0.015)
2023	0.433***	(0.015)
Midwest *(excluded region: Northeast)*	−0.140***	(0.019)
South	−0.114***	(0.018)
West	−0.042**	(0.018)
Constant	0.277***	(0.043)
Observations	7395	
Adjusted *R*-squared	0.168	

Table shows the results of an individual-level linear regression using 2020-2023 MEPS data on all individuals with any reported mental health service appointments. The dependent variable is the share of a patient's mental health visits conducted via telehealth. Regressions are weighted using survey weights. Standard errors are robust.

^*^ *P* < .10, **P < .05, ***P < .01.

We also show that telehealth is more common for patients who receive more frequent mental health treatment and who receive their care from an outpatient, rather than office-based provider. Patients without health insurance, older patients, and those without college education are less likely to have mental health visits via telehealth.

## Discussion

Our results show that despite the rapid expansion of telehealth use in mental health treatments, rural residents remain significantly less likely to have seen a mental health provider in the last year, even after controlling for differences in self-reported mental health, and demographic and socio-economic differences. In fact, the gap between non-metro and large central metro residents appears to have grown since 2020. While stigma likely plays a role,^14^ this result raises important questions regarding the ability of telehealth to improve the gap in access to mental health care for rural relative to urban residents.

We also show that publicly insured patients, who were more than 5 percentage points more likely than privately insured patients to have seen a mental health care provider in 2015, are only 2 percentage points more likely to have seen a mental health care provider in 2024. This narrowing raises important questions about whether the expansion of telehealth benefitted publicly and privately insured patients equally and about telehealth's role in promoting equitable use of mental health services.

In our analyses, non-metro residents are significantly less likely to have had a telehealth appointment, even after controlling for differences in mental health care use and access to internet. In other words, our results show that lower income, demographic and insurance differences, lower mental health care utilization, and worse access to the internet are not the main correlates of significantly lower telehealth utilization for non-metro residents.

We show that for patients who did receive mental health treatments, telehealth use is associated with socio-economic and demographic variables, with the frequency of mental health treatments, and also with whether a patient self-pays for their treatment.

The correlation between self-pay and telehealth use indicates that providers who see a larger share of patients via telehealth can benefit from higher rates per session. In a field of health care with a long documented preference for self-pay—for example, in 2009-2010, 42% of psychiatrists accepted Medicaid and 55% accepted Medicare or private insurance^15^—there are 2 potential explanations for this result: First, it is possible that telehealth is disproportionally attractive to providers with lower willingness to accept lower insurance reimbursement, perhaps because of patient selection, so that the composition of providers who do and do not perform a large share of their care via telehealth is different. Second, it raises the possibility that telehealth might exacerbate a preference of mental health providers toward self-payment or insurance forms that lead to higher reimbursement. Providers who practice entirely remotely may be more likely to work independently and thus not have staff to submit insurance claims and process reimbursements, making it more attractive to seek out self-paying patients. Also, given that the quantity of mental health services demanded exceeds the quantity supplied, telehealth allows providers to accept patients outside of their area of residence, thus making it easier to be selective among patients.^16^

While we are not able to directly investigate the mechanisms underlying the differential rate of self-pay, we caution that for rural residents, who are on average older and have lower income, this correlation might imply that telehealth does not in fact allow them to find a provider more easily. This interpretation is consistent with existing evidence that in a setting without differences in reimbursement rates across patients, rurality does not significantly predict virtual mental health care use once broadband access is accounted for.^17^

## Limitations

Several limitations of this study merit consideration and provide opportunities for future research. First, our evidence linking telehealth expansion to reduced access for publicly insured and rural patients is descriptive and correlational rather than causal. In particular, our analyses do not provide causal evidence on the factors underlying the changes observed in the probability of seeing a mental health professional over time. Second, while we theorize that telehealth enables providers to become more selective in accepting lower-reimbursement patients, our data do not directly observe provider selection behavior; the self-payment findings in Table 2 are consistent with this mechanism but do not confirm it. Third, our analyses are limited by the availability of key variables in the NHIS and MEPS data over time. For example, we can only control for internet access as a confounder with 2022-2024 NHIS data due to data availability constraints, which excludes the potentially important telehealth adoption period of 2020-2021. We are also limited to investigating the use of telehealth only since 2020, and cannot separately identify the use of telehealth at mental health visits with the NHIS data.

## Conclusion

Limited success in attracting more mental health providers to shortage areas has made increased telehealth usage an attractive solution to narrow gaps in access for rural residents. While telehealth clearly has the potential to make access to health care more equitable, in particular for patients who live in areas without adequate access to providers in-person, we provide evidence and arguments that telehealth expansion alone may not be sufficient to address gaps in access, at least in mental health care. Our descriptive results suggest that telehealth introduces a service model that eases access overall but, like existing models, may disproportionately benefit urban and more affluent residents rather than increasing overall provider availability.

We show that the gap in access to mental health providers for non-metro residents is large (roughly 5 percentage points) and growing. At the same time, telehealth service use is 8–9 percentage points lower for non-metro residents, even after accounting for differences in mental health care utilization and internet access. In addition to demographic and socio-economic factors, self-payment is a significant predictor of telehealth utilization in mental health care. We argue that this result can help explain why it may be more difficult for non-metro residents, who have disproportionately lower incomes and are more likely to rely on public insurance, to access telehealth services. This is critical to consider if telehealth is to be harnessed as a tool to effectively reduce disparities in access to care. As long as the national demand for mental health services exceeds the provider supply, policy-makers should be conscious that telehealth usage is lower for patients who already face higher barriers to health care access. Any efforts to promote telehealth should therefore be coupled with targeted efforts to make it easier for providers to serve disadvantaged patients, including equitable reimbursement and lower administrative barriers to billing public and private insurance. Without deliberate policy reform, telehealth expansion should not be viewed as a substitute to increase the provider supply nationwide, especially in provider shortage areas.

## Supplementary Material

qxag241_Supplementary_Data
