# Supplementary material for: Mental health care access in rural communities amid the expansion of telehealth
Source: Health Aff Sch. 2026 Sep 4;4(9):qxag241. doi: 10.1093/haschl/qxag241 (PMC13602415; doi:10.1093/haschl/qxag241)
Supplement: Supplementary file 1 [file qxag241_supplementary_data.zip › Online Appendix-1.docx]

## Online Appendix

## Appendix Table 1 — Summary Means, 2020-2024 NHIS Data

|  | % | N |
| --- | --- | --- |
| Share of Individuals with Telehealth Appt | 28.32 | 150852 |
| Share of Individuals who saw Mental Health Professional | 12.07 | 150852 |
| Share Ever Experience Depression | 46.87 | 147656 |
| Share Ever Told Anxiety Disorder | 17.37 | 150608 |
| No Health Insurance | 7.40 | 150852 |
| Private Health Insurance | 31.44 | 150852 |
| Medicare FFS Insurance | 12.47 | 150852 |
| Medicare Advantage Insurance | 10.54 | 150852 |
| Medicaid Insurance | 88.77 | 150852 |
| Share of Individuals with Usual Place for Medical Care | 14.13 | 150852 |
| Age 18-30 | 30.02 | 150852 |
| Age 31-50 | 25.51 | 150852 |
| Age 51-65 | 30.34 | 150852 |
| Age 65 or older | 54.29 | 150852 |
| Female | 44.92 | 150852 |
| Married | 74.92 | 150852 |
| White | 11.01 | 150852 |
| Black | 5.84 | 150852 |
| Asian | 8.22 | 150852 |
| All Other Races | 13.77 | 150852 |
| Hispanic | 8.81 | 150852 |
| No HS Degree | 24.95 | 150852 |
| HS Degree | 66.23 | 150852 |
| At Least Some College | 28.32 | 150852 |
| Income:Pov Threshold Ratio < 1 | 9.96 | 150852 |
| Income:Pov Threshold Ratio 1-2 | 17.47 | 150852 |
| Income:Pov Threshold Ratio 2-3 | 16.31 | 150852 |
| Income:Pov Threshold Ratio >3 | 56.25 | 150852 |
| Non-Metro | 16.68 | 150852 |
| Medium to Small Metro | 30.65 | 150852 |
| Large Fringe Metro | 22.91 | 150608 |
| Large Central Metro | 29.76 | 150852 |

*Notes*: Appendix Table 1 provides a summary of our sample using 2020-2024 NHIS data. We report the percentage for all categorical variables, as well as the number of non-missing observations.

## Appendix Table 2 — Predicting Use of Mental Health Professional

|  | Dependent Variable: Saw a Mental Health Professional in the Last 12 Months  (1 = yes, 0 = no) | | | |  |
| --- | --- | --- | --- | --- | --- |
|  | Coeff. | Std. err. | Coeff. | Std. err. | |
| Ever Experience Depression | 0.0958*** | (0.0020) | 0.1530*** | (0.0018) | |
| Ever Told Anxiety Disorder | 0.2873*** | (0.0039) |  |  | |
| No Insurance *(excluded: Other Ins.)* | -0.0579*** | (0.0037) | -0.0801*** | (0.0032) | |
| Public Health Insurance | 0.0198*** | (0.0025) |  |  | |
| Public Insurance # 2015 |  |  | 0.0559*** | (0.0069) | |
| Public Insurance # 2016 |  |  | 0.0576*** | (0.0072) | |
| Public Insurance # 2017 |  |  | 0.0628*** | (0.0068) | |
| Public Insurance # 2018 |  |  | 0.0648*** | (0.0055) | |
| Public Insurance # 2020 |  |  | 0.0460*** | (0.0047) | |
| Public Insurance # 2021 |  |  | 0.0374*** | (0.0045) | |
| Public Insurance # 2022 |  |  | 0.0380*** | (0.0048) | |
| Public Insurance # 2023 |  |  | 0.0166*** | (0.0048) | |
| Public Insurance # 2024 |  |  | 0.0199*** | (0.0047) | |
| Has a Usual Place for Care | 0.0225*** | (0.0030) | 0.0343*** | (0.0026) | |
| Age 31-50 *(excluded cat: 18-30)* | -0.0140*** | (0.0033) | -0.0110*** | (0.0029) | |
| Age 51-65 | -0.0573*** | (0.0033) | -0.0574*** | (0.0029) | |
| Age 65 or older | -0.0985*** | (0.0033) | -0.1167*** | (0.0030) | |
| Female | 0.0112*** | (0.0019) | 0.0240*** | (0.0017) | |
| Married | -0.0193*** | (0.0020) | -0.0305*** | (0.0017) | |
| White (excluded cat: Other Races) | 0.0032 | (0.0037) | 0.0078** | (0.0035) | |
| Black | -0.0047 | (0.0048) | -0.0188*** | (0.0043) | |
| Asian | -0.0457*** | (0.0047) | -0.0566*** | (0.0043) | |
| Hispanic | -0.0180*** | (0.0031) | -0.0278*** | (0.0026) | |
| HS Degree *(excluded: No HS Degree)* | 0.0013 | (0.0033) | -0.0017 | (0.0029) | |
| At Least Some College | 0.0387*** | (0.0033) | 0.0352*** | (0.0029) | |
| Income:Pov Threshold Ratio 1-2 *(excluded: < 1)* | -0.0132*** | (0.0040) | -0.0220*** | (0.0034) | |
| Income:Pov Threshold Ratio 2-3 | -0.0173*** | (0.0041) | -0.0277*** | (0.0035) | |
| Income:Pov Threshold Ratio >3 | 0.0005 | (0.0040) | -0.0142*** | (0.0034) | |
| Non-metro # 2020 (excluded cat: Large Central Metro) | -0.0421*** | (0.0056) |  |  | |
| Non-metro # 2021 | -0.0395*** | (0.0057) |  |  | |
| Non-metro # 2022 | -0.0507*** | (0.0059) |  |  | |
| Non-metro # 2023 | -0.0594*** | (0.0058) |  |  | |
| Non-metro # 2024 | -0.0574*** | (0.0053) |  |  | |
| Small to Medium Metro | -0.0266*** | (0.0025) |  |  | |
| Large Fringe Metro | -0.0165*** | (0.0027) |  |  | |
| *(continued on next page)* |  |  |  |  | |
|  |  |  |  |  | |
| 2016 (excluded: 2015) |  |  | 0.0052 | (0.0058) | |
| 2017 |  |  | 0.0041 | (0.0058) | |
| 2018 |  |  | 0.0129** | (0.0051) | |
| 2020 | 0.0000 | (.) | 0.0149*** | (0.0049) | |
| 2021 | 0.0033 | (0.0031) | 0.0247*** | (0.0050) | |
| 2022 | 0.0147*** | (0.0032) | 0.0370*** | (0.0052) | |
| 2023 | 0.0211*** | (0.0032) | 0.0531*** | (0.0052) | |
| 2024 | 0.0282*** | (0.0032) | 0.0607*** | (0.0052) | |
| Midwest *(excluded region: Northeast)* | -0.0128*** | (0.0032) | -0.0141*** | (0.0028) | |
| South | -0.0204*** | (0.0028) | -0.0209*** | (0.0025) | |
| West | -0.0046 | (0.0031) | -0.0083*** | (0.0028) | |
| Constant | 0.0511*** | (0.0073) | 0.0362*** | (0.0073) | |
| Observations | 147489 | | 208322 | | |
| Adjusted R-squared | 0.208 | | 0.105 | | |

*Notes*: Appendix Table 2 shows the regressions underlying Figure 2. All regressions are based on 2015-2024 NHIS data, depending on availability of individual variables. Consequently, the two regressions are based on different samples. Regressions are weighted using survey weights. Standard errors are robust. * 𝑝 < 0.10, ** 𝑝 < 0.05, *** 𝑝 < 0.01.

## Appendix Table 3 — Summary Means of Patients with Mental Health Care Visits, 2020-2023 MEPS Data

|  |  | Sample Mean (SD) |
| --- | --- | --- |
| Share of Appointments via Telehealth |  | 0.29 |
| No OOP Payment |  | 0.46 |
| Some OOP Payment |  | 0.41 |
| Fully Self-Paid |  | 0.13 |
| Number of MH Visits |  | 12.85(17.21) |
| Share of MH Visits with Psychologist |  | 0.16 |
| Share of MH Visits with Psychiatrist |  | 0.26 |
| Share of MH Visits with Counselor |  | 0.35 |
| Share of MH Visits Office-Based |  | 0.91 |
| No Health Insurance |  | 0.02 |
| Private Health Insurance |  | 0.50 |
| Medicare FFS Insurance |  | 0.15 |
| Medicare Advantage Insurance |  | 0.08 |
| Medicaid Insurance |  | 0.19 |
| Age 18-30 |  | 0.23 |
| Age 31-50 |  | 0.38 |
| Age 51-65 |  | 0.24 |
| Age 65 or older |  | 0.14 |
| Female |  | 0.64 |
| Married |  | 0.38 |
| White |  | 0.80 |
| Black |  | 0.12 |
| Asian |  | 0.01 |
| All Other Races |  | 0.07 |
| Hispanic |  | 0.13 |
| HS Degree |  | 0.20 |
| At Least Some College |  | 0.70 |
| Poor |  | 0.17 |
| Near Poor |  | 0.05 |
| Low Income |  | 0.11 |
| Middle Income |  | 0.25 |
| High Income |  | 0.42 |
| Observations |  | 7500 |

*Notes*: Appendix Table 3 provides a summary of the individuals with at least one mental health appointment using 2020-2023 MEPS data. We show the percentage for each categorical variable and the mean (SD) for each continuous variable. s
